# BGLF2 Increases Infectivity of Epstein-Barr Virus by Activating AP-1 upon *De Novo* Infection

**DOI:** 10.1128/mSphere.00138-18

**Published:** 2018-04-25

**Authors:** Natsuno Konishi, Yohei Narita, Fumiya Hijioka, H. M. Abdullah Al Masud, Yoshitaka Sato, Hiroshi Kimura, Takayuki Murata

**Affiliations:** a Department of Virology, Nagoya University Graduate School of Medicine, Nagoya, Japan; b Department of Virology and Parasitology, Fujita Health University School of Medicine, Toyoake, Japan; c Division of Infectious Disease, Department of Medicine, Brigham & Women’s Hospital, Harvard Medical School, Boston, Massachusetts, USA; UNC—Chapel Hill

**Keywords:** AP-1, BGLF2, BRLF1, EBV, cell signaling

## Abstract

Epstein-Barr virus (EBV) is a human gammaherpesvirus that causes infectious mononucleosis and several malignancies, such as endemic Burkitt lymphoma and nasopharyngeal carcinoma. Herpesviruses carry genes that can modify cell functions, including transcription and ubiquitination, thereby facilitating viral growth and survival in infected cells. Using a reporter screening system, we revealed the involvement of several EBV gene products in such processes. Of these, BGLF2 activated the AP-1 signaling pathway through phosphorylation of p38 and c-Jun N-terminal kinase (JNK). Knockout of the BGLF2 gene did not affect viral gene expression and viral genome DNA replication, but resulted in marked reduction of progeny titer. We also found that the BGLF2 disruption resulted in significant loss of infectivity upon *de novo* infection. Interestingly, expression of a binding partner, BKRF4, repressed the activation of AP-1 by BGLF2. These results shed light on the physiological role of the tegument protein BGLF2.

**IMPORTANCE** Epstein-Barr virus (EBV), an oncogenic gammaherpesvirus, carries ~80 genes. While several genes have been investigated extensively, most lytic genes remain largely unexplored. Therefore, we cloned 71 EBV lytic genes into an expression vector and used reporter assays to screen for factors that activate signal transduction pathways, viral and cellular promoters. BGLF2 activated the AP-1 signaling pathway, likely by interacting with p38 and c-Jun N-terminal kinase (JNK), and increased infectivity of the virus. We also revealed that BKRF4 can negatively regulate AP-1 activity. Therefore, it is suggested that EBV exploits and modifies the AP-1 signaling pathway for its replication and survival.

## Abstract (precis)

Epstein-Barr virus (EBV), an oncogenic gammaherpesvirus, carries ~80 genes. While several genes have been investigated extensively, most lytic genes remain largely unexplored. Therefore, we cloned 71 EBV lytic genes into an expression vector and used reporter assays to screen for factors that activate signal transduction pathways, viral and cellular promoters. BGLF2 activated the AP-1 signaling pathway, likely by interacting with p38 and c-Jun N-terminal kinase (JNK), and increased infectivity of the virus. We also revealed that BKRF4 can negatively regulate AP-1 activity. Therefore, it is suggested that EBV exploits and modifies the AP-1 signaling pathway for its replication and survival.

## INTRODUCTION

Epstein-Barr virus (EBV), a gammaherpesvirus, is a ubiquitous virus that infects >90% of adults. Primary EBV infection during infancy is usually asymptomatic; however, it occasionally causes infectious mononucleosis upon primary infection during or after adolescence. Once infected, EBV can never be eliminated because of its sophisticated silent mode of latent infection. The long-term presence of EBV can result in the formation of some types of cancers, such as Burkitt lymphoma, Hodgkin lymphoma, nasopharyngeal carcinoma, and gastric carcinoma (1, 2).

A portion of EBV in infected cells can switch from latent to lytic infection, an active mode in which all the viral genes are expressed, viral DNA is replicated, and progeny virions are eventually produced (3). The precise mechanism of this switch *in vivo* remains unclear; however, reactivation can be induced in cell culture by chemical or biological agents or exogenous expression of viral immediate early (IE) genes coding for BZLF1 (the Zta, Z, Zebra, and EB1 genes) and/or BRLF1 (the Rta and R genes). Expression of the IE genes induces viral early (E) genes, including the DNA polymerase catalytic subunit or protein kinase. The E genes catalyze replication of viral genomic DNA, followed by production of viral late (L) genes. The L genes include genes coding for capsid, tegument, and glycoproteins, and these proteins contribute to the morphogenesis of progeny virus particles that are capable of infecting new cells (1, 4).

Herpesviruses adeptly exploit host cell functions, such as transcription, replication, cell death, and membrane biogenesis. For example, the herpes simplex virus (HSV) VP16 protein functions as an efficient transcriptional coactivator of viral IE genes by binding to the host cell factor/octamer-binding protein 1 (HCF/OCT-1) complex (5). The EBV BPLF1 tegument gene, encoding a deubiquitinase, targets several signaling molecules and replication factors and increases viral amplification (6–9). EBV EBNA2 interacts with recombination signal binding protein suppressor of hairless (RBPJκ) and activates Notch signaling (10). Herpesvirus nucleocapsids are transported from the nucleus to the cytoplasm and are guided by several viral capsid, tegument, and glycoproteins to exit the virion at the plasma membrane (11).

EBV carries approximately 80 genes, many of which require further characterization. Thus, an all-encompassing, efficient, and comparative analysis is necessary to elucidate the functions of each EBV gene. To investigate the involvement of EBV genes in gene expression processes, we cloned 71 EBV lytic genes and evaluated them in reporter assays. We found that BGLF2 markedly induced activator protein 1 (AP-1)-dependent transcription, possibly by binding to p38 and c-Jun N-terminal kinase (JNK) mitogen-activated protein kinases (MAPKs). Infectivity with a BGLF2 knockout virus was significantly reduced upon *de novo* infection of B cells, indicating that BGLF2 reinforced EBV infection as a tegument protein. Our results reveal a comprehensive picture of EBV-regulated transcriptional gene expression.

## RESULTS

### Screening of EBV genes that can induce transcription.

**FIG 1:**
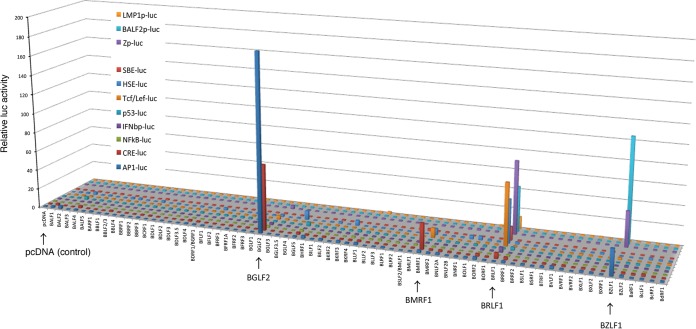
Screening for EBV-encoded transcriptional activators. HEK293T cells were transfected with the indicated reporter plasmid encoding firefly luciferase (10 ng) and an expression vector containing the HA-tagged EBV gene (200 ng). An internal control *Renilla* luciferase vector (null-RL) (10 ng) was cotransfected in order to monitor transfection efficiency. Relative luciferase activity is shown after normalization to RL activity. The luciferase activity of the control pcDNA vector was set as 1.

We prepared expression plasmids for 71 EBV lytic genes and transfected them into HEK293T cells, along with firefly luciferase reporter and *Renilla* luciferase (internal control) vectors. We here used eight reporter vectors of major host signal pathways and three additional reporters with viral promoters, including IE (Zp-luc), E (BALF2p-luc), and latent (LMP1p-luc) promoters as listed in Fig. 1. Interestingly, only a few EBV genes could activate these reporters (Fig. 1). An EBV tegument protein, BGLF2, activated transcription from the AP-1- and cAMP response element (CRE)-dependent promoter (AP-1-luc and CRE-luc, respectively), as reported recently (12). The DNA polymerase processivity factor, BMRF1, activated the CRE-luc and other reporters (T-cell factor/lymphoid enhancer factor-luciferase [TCF/LEF-luc] and heat shock element-luciferase [HSE-luc]), although the activations were weak. Involvement of BMRF1 in transcription has been reported previously (13–16). Two viral IE genes, coding for BRLF1 and BZLF1, play important roles in transcription; however, their modes of activation differ. BZLF1 activated AP-1-luc reporter via direct binding of BZLF1 to a consensus AP-1 site (17); BRLF1 did not activate the reporter. BRLF1 activated TCF/LEF-, HSE-, and Smad-binding element (SBE)-dependent promoters, whereas BZLF1 did not. Both BZLF1 and BRLF1 strongly induced transcription from viral lytic promoters (Zp-luc and BALF2p-luc), although only BRLF1 enhanced transcription from the proximal LMP1 promoter (LMP1p-luc). Our results showing induction of LMP1 by BRLF1 corroborated the results reported in a previous study (18).

### Activation of AP-1-dependent transcription by BGLF2.

**FIG 2:**
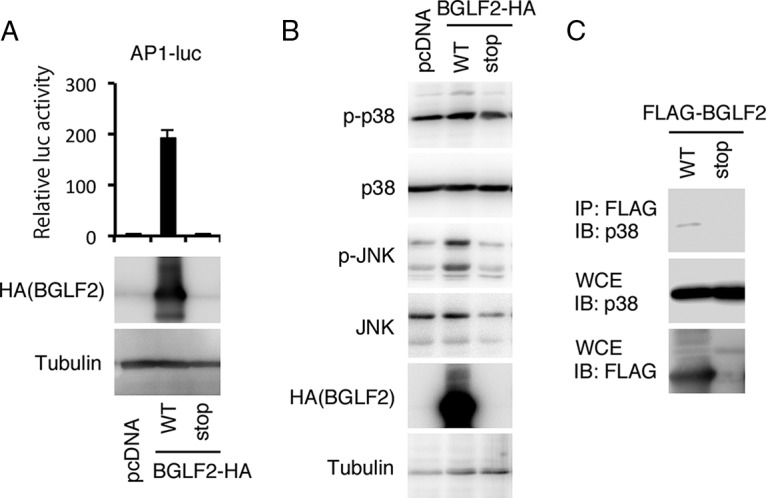
BGLF2 activates AP-1-dependent transcription. (A) Effect of BGLF2 on the AP-1-luc reporter. Luciferase assays were carried out as described for Fig. 1. The BGLF2-HA stop mutant serves as a negative control since it has a stop codon in the N-terminal part of the protein (the same mutation as the dBGLF2stop knockout virus in Fig. 3). The means ± standard deviations (SD) from three independent transfections are shown. Protein levels were also assessed by IB. (B) Effect of BGLF2 overexpression on phosphorylation of p38 and JNK. HEK293T cells were transfected with the indicated BGLF2 expression vectors and subjected to IB. (D) Association of BGLF2 with p38 MAPK. HEK293T cells were transfected with the indicated BGLF2 vectors and harvested for IP using an anti-FLAG antibody, followed by IB.

The EBV tegument protein, BGLF2, can potently enhance AP-1-responsive promoters (Fig. 1) as reported by Liu and Cohen (12). When wild-type (WT) BGLF2 markedly induced AP-1-dependent transcription, the BGLF2 stop mutant (BGLF2 with a stop codon in the N-terminal part of the open reading frame [ORF]) failed to induce the reporter (Fig. 2A).

We next examined the effect of BGLF2 overexpression in HEK293T cells on the phosphorylation of MAPKs. Phosphorylation of p38 and JNK was significantly upregulated by WT BGLF2 (Fig. 2B), as reported (12), although the increase of p38 phosphorylation was not very clear. Therefore, as reported previously by Liu and Cohen, BGLF2 activates AP-1-dependent transcription by inducing phosphorylation of p38 and JNK MAPKs.

Next, we examined the interaction between BGLF2 and p38 MAPK by immunoprecipitation. The BGLF2 WT showed an interaction with p38; in contrast, no interaction was observed in the BGLF2 stop mutant (Fig. 2C). We do not know why, but we could not clearly observe BGLF2’s interaction with JNK.

### Augmentation of infectivity by BGLF2.

**FIG 3:**
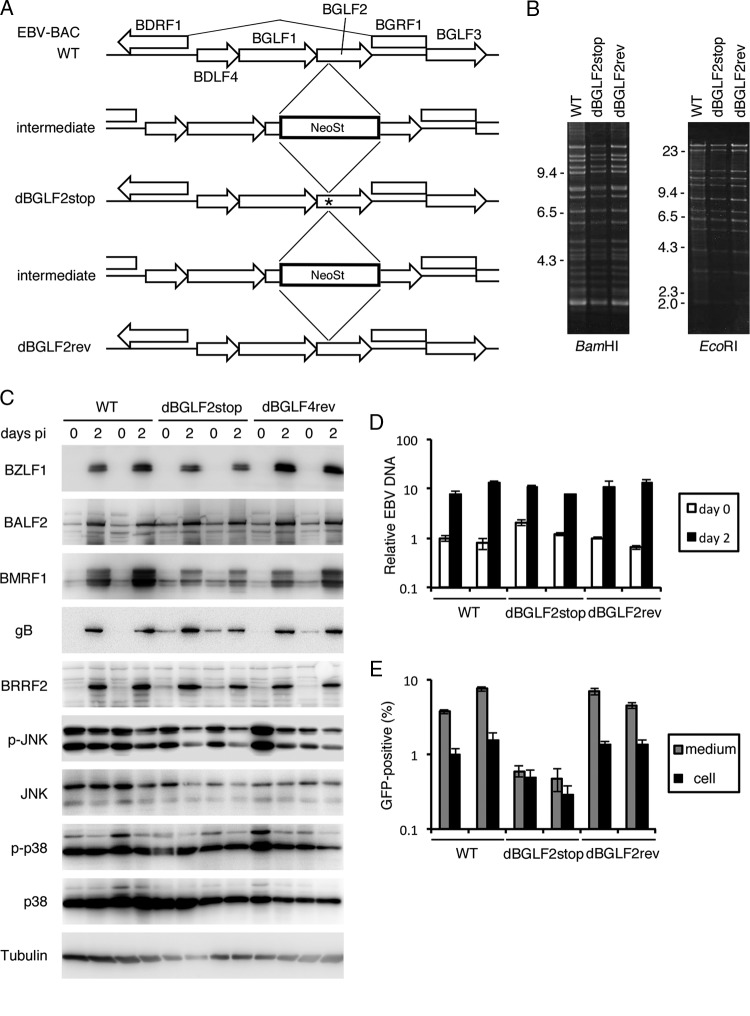
Disruption of the BGLF2 gene decreased infectious progeny production. (A) Schematic representation of the EBV-BAC recombination procedures. The asterisk in the dBGLF2stop strain indicates a stop codon in the viral genome. (B) Recombinant EBV-BAC genomes were digested with BamHI or EcoRI and electrophoresed on an agarose gel. (C) Protein expression in the recombinant viruses. HEK-293 cell clones that harbor the latent recombinant B95-8 EBV-BAC genome constructed above were transfected with the BZLF1 expression vector by electroporation. Cells were harvested at 0 and 2 days after transfection and subjected to IB. For each strain (WT, dBGLF2stop, and dBGLF2rev), the results from two representative clones are shown. (D) Lytic viral DNA synthesis of the recombinant viruses. Cells transfected as described for panel C were harvested at 0 and 2 days after transfection and subjected to qPCR to detect the EBV DNA and genomic DNA levels in the host cells. The means ± SD from three independent biological replicates are shown after normalization to the value of the host control. The day 0 value from one of the WT samples was set as 1. (E) Infectious progeny levels produced from the recombinant viruses. Cells were transfected as described for panel C, and after 3 days, cell-associated and cell-free EBV particles were titrated with Akata(−) cells by determining the GFP-positive ratio using fluorescence-activated cell sorting (FACS). The means ± SD from three independent biological replicates are shown.

We generated a BGLF2-knockout virus using the EBV-bacterial artificial chromosome (BAC) system (Fig. 3A). The marker cassette (NeoSt) was inserted into the N-terminal portion of BGLF2 to prepare an intermediate. Then dBGLF2stop was formed by replacing the cassette with the sequence containing a stop codon (*). This stop codon is the same mutation we used in the Fig. 2. Next, the cassette was again inserted and replaced with the WT sequence to make a revertant strain (dBGLF2rev). To confirm the integrity, these recombinant EBV-BAC genomes were digested with BamHI or EcoRI and electrophoresed on an agarose gel (Fig. 3B). Identical band patterns among the three strains indicated that the recombinant genomes did not carry obvious deletions or insertions. Sequence analysis confirmed that the dBGLF2stop mutant contained a stop codon as intended, while WT and dBGLF2rev did not (not shown). After transfecting the EBV-BAC DNAs into HEK293 cells, hygromycin-resistant and green fluorescent protein (GFP)-positive cell clones were isolated, in which recombinant EBVs were latently maintained.

Using the EBV-positive HEK293 cell lines, protein expression (Fig. 3C), viral DNA synthesis (Fig. 3D), and infectious progeny production (Fig. 3E) were compared. The cell clones were transfected with the BZLF1 expression vector to induce the lytic cycle and harvested on days 0 and 2 for immunoblot (IB) analysis (Fig. 3C). Two HEK293 clones were used for each strain, to ensure reproducibility. Although clonal variations were observed, the expression levels of the viral proteins (BZLF1, BALF2, BMRF1, gB, and BRRF2) were almost comparable among the WT, dBGLF2stop, and dBGLF2rev strains (Fig. 3C). The levels of phosphorylated JNK were marginally lower in the dBGLF2stop strain on day 2 (Fig. 3C). The viral DNA levels were similar among the WT, dBGLF2stop, and dBGLF2rev strains (Fig. 3D), indicating that the BGLF2 knockout virus synthesized the viral genome as efficiently as the WT and dBGLF2rev viruses. On the other hand, extracellular production of infectious viral progeny was significantly impaired in the BGLF2 knockout mutant by approximately 1 order of magnitude or more (Fig. 3E, gray). Cell-associated infectious viruses were also decreased (Fig. 3E, black). These data showed that disruption of BGLF2 caused inefficient maturation of infectious progeny and extracellular excretion.

**FIG 4:**
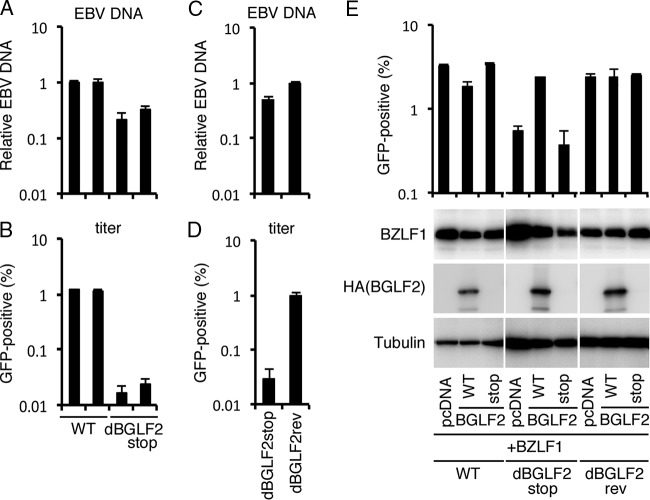
Requirement of BGLF2 for efficient virion production and *de novo* infection. (A) Knockout of BGLF2 caused reductions in progeny virus production and infectivity of the virus particles. Cells were transfected with the BZLF1 expression vector and the supernatants were harvested after 3 days. A portion of each supernatant sample was treated with Turbo DNase, and DNase-resistant DNA was purified for qPCR to determine viral DNA levels. The means ± SD from three independent biological replicates are shown. (B) The remaining supernatant sample was inoculated with Akata(−) cells. After 2 days, GFP positivity was determined by FACS. The means ± SD from three independent biological replicates are shown. (C and D) As in panels A and B, a portion of virus solution was subjected to qPCR (C) after DNase treatment, and virus particles in the rest of the sample were titrated by infection of Akata(−) cells, followed by FACS analysis (D). (E) Exogenous expression of WT BGLF2 restored the impaired infectivity of the knockout. HEK-293 cells latently infected with each recombinant strain of EBV (WT, dBGLF2stop, and dBGLF2rev) were transfected with the BZLF1 expression vector along with the WT or mutated form of BGLF2 expression vector as indicated. After 3 days, supernatants were harvested and inoculated with Akata(−) cells. After 2 days, GFP positivity was determined by FACS. The means ± SD from three independent biological replicates are shown. The protein levels were evaluated by IB.

Next, we quantified the viral DNA genome in the extracellular virions (Fig. 4A). Two HEK293 cell lines latently harboring WT or dBGLF2stop virus were lytically induced by BZLF1 transfection, the culture medium was collected on day 3, and cell debris was cleared by centrifugation. Naked DNA and viral DNA incorporated into imperfect virions in the supernatant were eliminated by the addition of Turbo DNase. The DNA was extracted and subjected to quantitative PCR (qPCR). Extracellular virion DNA levels in the knockout were impaired to 20 to 30% compared with those in the WT (Fig. 4A). Infectious virus particles in the same samples were titrated by infecting Akata(−) cells (Fig. 4B). GFP positivity was significantly lower in the dBGLF2stop strain by an order of magnitude or more (Fig. 4B). To confirm the result, the same experiment was repeated using knockout and revertant strains (Fig. 4C and D). DNase-resistant viral DNA of the stop mutant was 49% of that of the WT (Fig. 4C), and virus infectivity of the stop sample was only 3% of that of the WT (Fig. 4D). These results suggest that the BGLF2 gene product not only increased the extracellular secretion of virus particles but also played an important role in increasing infectivity upon *de novo* infection. This is not surprising, because BGLF2 protein is in the tegument component (19) and can enhance BZLF1 transcription by AP-1 activation (12).

The complementation assay was carried out to examine the effect of BGLF2 disruption further (Fig. 4E). HEK293 cell lines latently infected with WT, dBGLF2stop, and dBGLF2rev viruses were transfected with expression vector(s) harboring BZLF1 with or without the WT or stop mutant form of BGLF2. The culture supernatant was collected after 3 days and titrated in Akata(−) cells. The reduced titer in the BGLF2 knockout (dBGLF2stop) was restored by WT BGLF2 transfection but not by the BGLF2 stop mutant (Fig. 4E). These data suggested that the reduced yield in the knockout was caused specifically by the absence of BGLF2.

**FIG 5:**
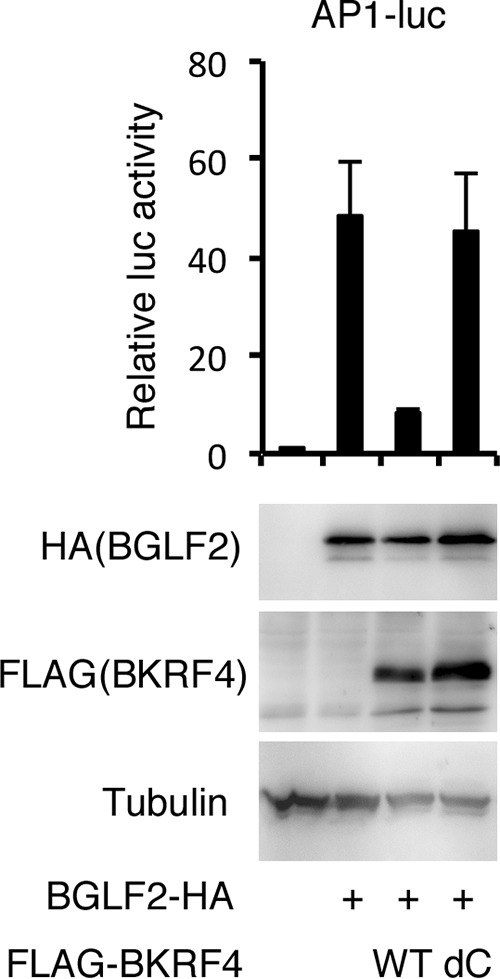
Effect of BKRF4 on AP-1-dependent transcriptional activation by BGLF2. HEK293T cells were transfected with the AP-1-luc reporter plasmid, an internal control *Renilla* luciferase vector (null-RL), with or without the BGLF2 expression vector and the WT or C-terminally truncated (dC) mutant of BKRF4, as indicated. Relative luciferase activity is shown after normalization to RL activity. The luciferase activity of the control pcDNA vector was set as 1. Protein levels were also assessed by IB.

Because we previously observed that a tegument protein, BKRF4, interacts with BGLF2 protein (20), we then examined if presence of BKRF4 could modify the activation of AP-1-dependent transcription by BGLF2. Interestingly, coexpression of WT BKRF4 clearly suppressed AP-1 activity induced by BGLF2 (Fig. 5, lane 3). It is important to note that when the dC mutant of BKRF4, which lacks 14 amino acids in the C terminus of BKRF4 and thus cannot interact with BGLF2 anymore (20), was cotransfected with BGLF2, the luciferase activity was not damaged (Fig. 5, lane 4). These data suggest that BKRF4 associates with BGLF2 to sequester and block the high AP-1 activity in the later period of the EBV lytic replication cycle, as BKRF4 is an L gene (20).

## DISCUSSION

EBV carries as many as 80 genes; however, most EBV genes remain largely unexplored. Moreover, most EBV gene analyses performed to date have employed a range of materials, strategies, and methods in different laboratories. The present study investigated EBV genes using a unified, comprehensive approach. We here cloned 71 EBV genes into an expression vector with a hemagglutinin (HA) tag on the C terminus and carried out large-scale reporter assays. The results corroborated previous reports in terms of the transcriptional activation by several EBV genes, such as those coding for BGLF2, BMRF1, and BZLF1, and also revealed several new transcription regulation systems used by EBV genes (Fig. 1).

Recently, a tegument protein of EBV, BGLF2, was shown to activate p38 and JNK signaling pathways and enhance AP-1-dependent transcription (12). The study showed that overexpression and knockdown of BGLF2 in EBV-positive cancer cells resulted in promotion and inhibition of the EBV lytic cycle, respectively (12). In this study, we prepared an EBV BGLF2 knockout strain and a repaired strain and compared the two viruses in HEK293 cells. The BGLF2 knockout virus produced viral proteins and synthesized its genomic DNA almost as efficiently as the WT and revertant strains (Fig. 3). However, disruption of BGLF2 reduced the amount of production of progeny virions in the supernatant and the infectivity of the virus particles after *de novo* infection (Fig. 3 and 4). We recently demonstrated that another tegument protein, BKRF4, associates with BGLF2 protein via its conserved C-terminal domain and that this interaction is important for progeny production and acquisition of efficient infectivity (20). It is of our significant interest that WT BKRF4 markedly inhibited BGLF2’s AP-1 activation, while the dC mutant did not (Fig. 5). This means that BKRF4 can stifle the BGLF2’s activity by interacting with BGLF2. We speculate that AP-1 activity may be beneficial for EBV gene expression upon *de novo* infection until late genes are expressed, but the high activity may not be needed in the following processes, such as morphogenesis of virus particles or egress. Therefore, BKRF4, which is expressed with L kinetics (20), may suppress the activity of BGLF2 by association with BGLF2 protein. Because both BKRF4 and BGLF2 are tegument proteins, they are incorporated together into the virus particles. Upon infection of naive cells, BGLF2 may somehow be released from BKRF4 and increase AP-1 activity in the cells in order to enhance expression of viral genes (Fig. 4B). Anyway, although the BGLF2 gene is conserved in all subfamilies of herpesviruses (as shown by the UL16 gene of HSV, UL94 gene of human cytomegalovirus [HCMV], and ORF33 of Kaposi’s sarcoma-associated herpesvirus [KSHV]), the BKRF4 gene is conserved only in gammaherpesviruses (ORF45 of KSHV). Therefore, this BGLF2-BKRF4 regulatory mechanism may be conserved only in the gammaherpesvirus subfamily. It is also possible that activation of MAPK signal pathway is conserved only for the EBV BGLF2 gene but not in the KSHV ORF33 because such a report is not available. Interestingly, KSHV ORF45 has been demonstrated to induce RSK/MAPK activity (21–26), while the EBV BKRF4 gene cannot (20). Thus, EBV and KSHV might have evolved in the different manner in terms of activation of the MAPK pathway in infected cells.

Two IE genes of EBV, coding for BZLF1 and BRLF1, are involved in transcriptional regulation of viral and cellular genes; however, their activities differ, depending on the promoter (Fig. 1). Both BZLF1 and BRLF1 induced activation of viral lytic promoters efficiently. The activation by BZLF1 was more restricted to viral lytic promoters (and AP-1-luc), whereas BRLF1 activated a relatively broad range of promoters (Fig. 1). This difference in target genes may be due to the different modes of action of the two transcriptional activators. BZLF1, which prefers to form homodimers, binds directly to the BZLF1-responsive element and activates transcription as a transcription factor.

On the other hand, BRLF1 (or its homologs in gammaherpesvirus group) efficiently interacts with various host transcription factors and mediates transcription as a cofactor (27–30). As shown in Fig. 1, we found that BRLF1 activated TCF/LEF-, HSE-, and SBE-dependent promoters, whereas BZLF1 did not. BRLF1 protein activates transcription either by (i) binding directly to DNA (Rta-responsive element [RRE]) (31), (ii) binding to host transcription factor(s) and acting as a transcriptional coactivator (28), or (iii) activating cell signaling pathways via an unknown mechanism (32). To investigate how BRLF1 activates such promoters, we used two mutant forms of BRLF1: the K156A mutant is unable to bind directly to the RRE (33), and the BRLF1 protein comprising 1 to 550 amino acids (d550) of 605 residues bound to DNA, but the C-terminal deletion resulted in loss of BRLF1 transcriptional activation function—likely because of the inability to interact with CREB-binding protein (CBP) (34–36). Interestingly, the TCF/LEF-, HSE-, and SBE-dependent reporters were induced by the K156A mutant almost as efficiently as by the WT; however, these promoters were not activated by the d550 mutant (not shown). We confirmed that expression levels were comparable between the WT and d550 and K156A mutants of BRLF1 (not shown). These results suggest that BRLF1 activates TCF/LEF-, HSE-, and SBE-dependent promoters without directly binding to the DNA, serving as a transcriptional cofactor. We speculate that BRLF1 recruits other transcriptional cofactors, such as CBP/p300, and activates transcription via the promoters to benefit promotion of EBV lytic cycle gene expression.

In this study, we conducted a comprehensive analysis of the EBV genes and identified several key viral components that function in transcriptional regulation. Further studies are required to determine the underlying mechanism of such regulation.

## MATERIALS AND METHODS

### Cell culture and reagents.

HEK293, HEK293 EBV-BAC, and HEK293T cells were maintained in Dulbecco’s modified Eagle’s medium (Sigma-Aldrich) supplemented with 10% fetal bovine serum (FBS). Akata(−) cells were cultured in RPMI 1640 medium (Sigma-Aldrich) containing 10% FBS. The antibodies against BZLF1, BRLF1, BMRF1, BALF2, BALF4, and BRRF2 have been reported previously (37–39). Anti-FLAG and anti-HA antibodies were purchased from Sigma-Aldrich. Antibodies against total and phosphorylated p38 MAPK and JNK and an anti-α/β-tubulin antibody were obtained from Cell Signaling. Horseradish peroxidase (HRP)-conjugated goat antibodies to mouse or rabbit IgG were purchased from Amersham Biosciences, Inc. Hygromycin B was purchased from Clontech.

### Plasmids.

The expression vector containing BZLF1 has been reported previously (40). For construction of EBV gene expression vectors, we prepared the following oligonucleotides: AATTCTACCCATACGATGTTCCAGATTACGCTTAAG and GATCCTTAAGCGTAATCTGGAACATCGTATGGGTAG. (The underlined nucleotides indicate the HA sequence.) The primers were annealed and inserted into the EcoRI and BamHI site of the pcDNA3.1(−) vector to prepare pcDNA-HA; next, the vector was digested with XhoI and EcoRI for linearization. The ORF sequences of the EBV genome were amplified from B95-8 EBV-BAC (41) by PCR using PrimeSTAR Max polymerase (TaKaRa) and the appropriate primers, with overlapping nucleotides. For example, to construct the BGLF2 expression vector, the primers **AAACGGGCCCTCTAGA**ATGGCATCCGCCGCGAACAG and **CGTATGGGTAGAATTC**ATAAGAATGTAAGACCTGAC were used, with the sequences in boldface representing the ends of the vector fragment. The linearized pcDNA-HA vector and each EBV ORF were linked using the In-Fusion cloning system (TaKaRa), according to the manufacturer’s instructions. After preparing the HA-tagged expression vectors, we verified that the sequences of the inserted EBV genes were identical to the sequences in the database (v01555.2). If a cloned EBV gene contained a mismatch, the mismatch was corrected by PCR. Mutagenesis of the BGLF2 expression vector or other gene products was performed by PCR using the appropriate primers and then confirmed by sequencing. AP-1-luc, CRE-luc, NF-κB-luc, TCF/LEF-luc, HSE-luc, SBE-luc, and null-RL (pGL4.70) were purchased from Promega. The beta interferon promoter (IFN-βp)-luc and p53-luc were gifts from K. Shimotohno and B. Vogelstein, respectively. The FLAG-tagged BKRF4 expression vectors (WT and dC), Zp-luc, BALF2p-luc, and LMP1p-luc (pLMP1/ED-L1-Fluc) have been reported previously (13, 20, 42, 43).

### Luciferase assays.

HEK293T cells were transfected with a firefly luciferase reporter plasmid, control *Renilla* luciferase plasmid (pRL-null), and an effector plasmid expressing an EBV gene using Lipofectamine 2000 (Thermo Fisher Scientific). After 24 h, cells were lysed and subjected to luciferase assays using the Promega dual-luciferase reporter assay system.

### Transfection, IB, and IP.

HEK293T cells were transfected with the indicated plasmid DNAs using Lipofectamine 2000 reagent (Thermo Fisher Scientific) or by electroporation using the Neon transfection system (Thermo Fisher Scientific). The total amount of plasmid DNA was standardized by the addition of an empty vector. Cells were washed with phosphate-buffered saline (PBS), harvested, and solubilized in sample buffer for IB, as described previously (40).

For immunoprecipitation (IP) experiments, HEK293T cells were transfected with the indicated plasmid DNA. At 24 h posttransfection, cells were solubilized in IP lysis buffer (20 mM Tris-HCl [pH 7.8], 150 mM NaCl, 0.5% NP-40, 1 mM EDTA, protease inhibitor cocktail [Complete mini; Roche], and phosphatase inhibitor cocktail tablets [PhoSTOP; Roche]), followed by sonication and centrifugation at 15,000 rpm for 5 min. The supernatants were mixed with anti-FLAG mouse antibody and protein G Sepharose 4 Fast Flow (GE Healthcare) and incubated at 4°C for >2 h with rotation. Immunocomplexes were washed four times with the IP lysis buffer. Samples were subjected to SDS-PAGE, followed by IB using the antibodies indicated in the figures. TrueBlot goat anti-rabbit/mouse IgG HRP-conjugated antibodies (EBioscience) were used as the secondary antibodies.

### EBV-BAC DNA genetic manipulation and cloning into HEK293 cells.

B95-8 EBV-BAC DNA was provided by W. Hammerschmidt (41). Homologous recombination was carried out in E. coli as described previously (40). To prepare recombinant viruses, a transfer DNA fragment for the first recombination was generated by PCR using the rpsL-neo vector (Gene Bridges) as the template and the following primers: Neo/St forward CAGGCCCGAGGTTCTCTTCACTAAGGCAGTCCAGGGGCCACACAGCCTGACTCTCATGTAGGCCTGGTGATGATGGCGGGATC and Neo/St reverse AGGGTTACCCCTAATCTCCACAGGCACCGCCTCACCCACTGCATCTGAGAATACCCCAAATCAGAAGAACTCGTCAAGAAGG. To obtain the insertion mutant (intermediate), kanamycin-resistant colonies were selected after recombination and verified by colony PCR using the following primers: GTGACCGTCTACATCAATGG and AGATCTGTGCAGGTGACTAC. The dBGLF2stop mutant was constructed by replacing the Neo/St cassette with a BGLF2 sequence containing a stop codon. Next, the Neo/St cassette was inserted once again to make an intermediate, and the cassette was replaced with the WT BGLF2 sequence to prepare the revertant strain. Electroporation of Escherichia coli was performed using Gene Pulser III (Bio-Rad), and EBV-BAC DNA was purified using NucleoBond Bac100 (Macherey-Nagel). Recombination was confirmed by PCR, sequence analysis, and electrophoresis of the BamHI- or EcoRI-digested viral genome.

HEK293 cells were transfected with the recombinant EBV-BAC DNA using Lipofectamine 2000 reagent, followed by culture on 10-cm dishes maintained using 150 µg/ml hygromycin B. After 2 weeks, GFP-positive, hygromycin-resistant cell colonies were cloned.

### Quantification of viral DNA synthesis.

Levels of viral DNA synthesis were determined using quantitative PCR (qPCR), as described previously (44). Briefly, cells were washed with PBS, lysed in lysis buffer with sonication, and treated with proteinase K. After deactivation of the proteinase, qPCRs were performed using the Fast Start universal probe master mix (Rox; Roche Applied Science). A standard curve determined using serial dilutions of DNA was used to quantify the amount of DNA. The probe and primers for detecting the viral genome were designed to target the BALF2-coding region.

### Virus titration by fluorescence-activated cell sorting analysis.

To induce lytic replication in HEK-293 cells carrying EBV-BAC, cells were transfected with pcDNABZLF1 using the Neon transfection system. After 3 days, cells and culture media were collected and subjected to centrifugation. Next, Akata(−) cells were infected with the virus solution for 3 h at room temperature with rotation. After 2 days, the cells were fixed with 1% formaldehyde, washed with PBS, and resuspended in PBS. GFP-positive cells were counted using the FACS Calibur G5 system (Becton, Dickinson), according to the manufacturer’s instructions.

### Quantification of extracellular virion DNA.

Extracellular virions were quantified using qPCR as described previously with some modifications (45). Briefly, virus stock was treated with Turbo DNase I (Thermo Fisher Scientific) for 1 h at 37°C. The reaction was stopped, and the mixture was incubated at 75°C for 10 min to inactivate the DNase. Then, the DNA was extracted using the DNeasy blood and tissue kit (Qiagen). Finally, the relative levels of viral DNA were quantified as described above.
